# Matrix Effects in a Fluid Catalytic Cracking Catalyst Particle: Influence on Structure, Acidity, and Accessibility

**DOI:** 10.1002/chem.201905867

**Published:** 2020-08-12

**Authors:** Marjolein E. Z. Velthoen, Alessandra Lucini Paioni, Iris E. Teune, Marc Baldus, Bert M. Weckhuysen

**Affiliations:** ^1^ Debye Institute for Nanomaterials Science Utrecht University Universiteitsweg 99 3584 CG Utrecht The Netherlands; ^2^ Bijvoet Center for Biomolecular Research Utrecht University Padualaan 8 3584 CH Utrecht The Netherlands

**Keywords:** acidity, binder effects, fluid catalytic cracking, matrix effects, spectroscopy

## Abstract

Matrix effects in a fluid catalytic cracking (FCC) catalyst have been studied in terms of structure, accessibility, and acidity. An extensive characterization study into the structural and acidic properties of a FCC catalyst, its individual components (i.e., zeolite H‐Y, binder (boehmite/silica) and kaolin clay), and two model FCC catalyst samples containing only two components (i.e., zeolite‐binder and binder‐clay) was performed at relevant conditions. This allowed the drawing of conclusions about the role of each individual component, describing their mutual physicochemical interactions, establishing structure‐acidity relationships, and determining matrix effects in FCC catalyst materials. This has been made possible by using a wide variety of characterization techniques, including temperature‐programmed desorption of ammonia, infrared spectroscopy in combination with CO as probe molecule, transmission electron microscopy, X‐ray diffraction, Ar physisorption, and advanced nuclear magnetic resonance. By doing so it was, for example, revealed that a freshly prepared spray‐dried FCC catalyst appears as a physical mixture of its individual components, but under typical riser reactor conditions, the interaction between zeolite H‐Y and binder material is significant and mobile aluminum migrates and inserts from the binder into the defects of the zeolite framework, thereby creating additional Brønsted acid sites and restoring the framework structure.

## Graphical abstract

**On the interplay between matrix and catalyst**: A combination of advanced characterization techniques reveals that while a freshly prepared spray‐dried fluid catalytic cracking catalyst appears as a physical mixture of its individual components, significant matrix effects actually come into play under industrial riser reactor conditions, leading to changing Brønsted and Lewis acidity, pore accessibility as well as various structural changes.

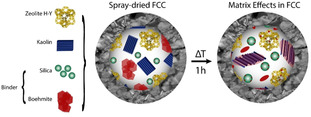

## Introduction

Zeolite‐based catalysts are employed on a large scale in many different industrial processes, such as crude oil refining (e.g., catalytic cracking, isomerization and aromatization reactions) and methanol‐to‐hydrocarbons (MTH).^1^, ^2^, ^3^ For optimal functionality, zeolites are often heterogeneously dispersed within a matrix and shaped into catalyst bodies. Typical matrix components include clays, such as kaolinite and attapulgite, and amorphous alumina or silica. These matrix components offer several important advantages, such as heat and attrition resistance, but also mechanical and chemical stability and proper accessibility to the acid sites. Moreover, such shaped catalyst bodies are easier to handle and recover from the reactor than zeolite powders.^4^, ^5^, ^6^ The influence of matrix components, however, can reach further than merely the physical advantages described above. Only recently, the academic literature has started to shift its focus from zeolite powders to full catalyst bodies with all the intrinsic complexity to investigate the influence of matrix components on the catalytic performance of the catalyst. This importance has, for example, been highlighted in a perspective article by Hargreaves and Munnoch.^5^ Matrix materials can have both beneficial or detrimental effects on the catalytic performance parameters, such as decreased or increased catalyst lifetime, acidity, pore accessibility, and product selectivity.^5^, ^6^, ^7^, ^8^, ^9^, ^10^

Fluid catalytic cracking (FCC) is an important catalytic technology in a crude oil refinery that employs such a complex and multi‐component zeolite‐based catalyst material.^11^, ^12^ In the FCC process, hot catalyst is mixed with vacuum gas oil (VGO) or heavy gas oil (HGO) and transferred to the riser reactor. In the order of seconds, the oil is cracked into smaller fragments at temperatures of approximately 550 °C. Due to the rapid formation of gaseous products, the mixture rises to the top of the reactor, where the products are stripped from the catalyst. The spent catalyst particles then travel further to the regenerator, where the catalyst is regenerated by burning the coke from the catalyst. It is then ready for reuse in the catalytic oil cracking.^12^

Crystalline microporous aluminosilicates of the faujasite (FAU) framework type (i.e., a stabilized version of zeolite Y) are the main responsible species for the cracking activity and selectivity in the FCC process. These zeolite domains are mixed with a binder (typically a silica/alumina phase) and a filler (typically a clay mineral) and, subsequently, spray‐dried to form spherical liquid‐like behaving catalyst bodies of 50–150 μm. The dilution of the active zeolite phase is highly necessary to prevent excessive gaseous product formation in the riser reactor,^6^, ^11^, ^13^ but the matrix components also play an important role in the catalytic performance of the shaped catalyst bodies.^14^

The overall reactivity of the FCC catalyst relies on the presence, strength and accessibility of acid sites.^11^, ^15^ The substitution of SiO_4_ tetrahedra with AlO_4_ creates a negatively charged zeolite framework. Counter ions in the form of protons can balance this negative charge, resulting in the formation of Brønsted acid sites in zeolite materials. The zeolite framework gives rise to various channels and cages of molecular dimensions, providing shape‐selective pores for the selective production of the desired products, including gasoline or lower olefins, such as propylene.^6^, ^16^ Lewis acid sites in the matrix, originating from aluminum species, are capable of pre‐cracking the long‐chain oil molecules, prior to entering the zeolite micropores.

Previous work from our research group on structure‐acidity relations in the FCC catalyst has mainly dealt with individual FCC catalyst particles and the visualization of Brønsted acid sites by chemical staining and subsequent confocal fluorescence microscopy.^17^, ^18^, ^19^, ^20^ Staining probe molecule reactions (i.e., acid catalyzed oligomerization reactions of styrene, thiophene, and furfuryl alcohol derivatives yielding fluorescent reaction products) have revealed the influence of different binders or clays on the amount and strength of different Brønsted acid sites in the catalyst body.^7^, ^9^, ^17^, ^18^, ^20^, ^21^, ^22^, ^23^, ^24^ Lewis acid sites are, however, more difficult to localize and visualize and have not yet received considerable attention. The nature of these sites is, therefore, not as understood as for Brønsted acid sites. By definition, Lewis acidity is an electron deficiency, originating from coordinatively unsaturated sites (CUS). Under ambient conditions, such sites often interact with water molecules, thereby saturating the sites and losing their Lewis acidic properties.^25^ Under reaction conditions, the temperature is higher (550 °C) and the catalyst is dehydrated, making the Lewis acid sites available for catalysis. It is, therefore, highly necessary to study acidic properties at similar temperatures to FCC reaction conditions to ensure the relevance and reliability of the study.

In this work, we have investigated whether the acidity in an FCC catalyst originates from the inherent acidic properties of the individual catalyst components (e.g., zeolite material), or whether synergistic or detrimental interactions occur between the different components within the catalyst body, thereby either creating or deleting acid sites. Such interactions are referred to as matrix effects. These results on matrix effects then lead to draw conclusions about the structural nature and location of all acid sites within the FCC catalyst. The FCC catalyst under study contains a faujasite zeolite (i.e., zeolite H‐Y), a kaolin clay and a silica/boehmite binder. An extensive comparison between the structural, physical, and acidic properties of the single components versus the fully shaped FCC catalyst is presented. Temperature‐programmed desorption (TPD) of NH_3_ and FTIR spectroscopy of adsorbed CO provide information on the acidic properties of the samples under study and reveal the synergistic effect of component interaction within the FCC catalyst. Next, structure‐acidity relations are established employing multiple quantum‐magic angle spinning‐nuclear magnetic resonance (MQ‐MAS‐NMR) spectroscopy, transmission electron microscopy (TEM), X‐ray diffraction (XRD), and Ar physisorption.

The investigation into matrix effects in FCC catalysts not only requires maximum exploitation of the characterization toolbox at hand, but also a well‐defined set of samples, comprising the individual catalyst components, the fully mounted spray‐dried FCC catalyst, but also spray‐dried samples containing only two out of three components (i.e., binder‐zeolite and binder‐clay combinations) to differentiate more clearly between the various interactions within the FCC catalyst material. Characterization of the acidic properties of the individual components allows for modeling the acidic properties of the spray‐dried samples. The comparison between model and experiment clearly visualizes synergistic and antagonistic effects as a result of component interaction. Importantly, this work considers all types of acid sites, pays equal attention to all FCC catalyst components, and, moreover, takes into account the reaction conditions of the FCC process. As a result, detailed structure‐acidity relationships for the entire FCC catalyst material are established, to the best of our knowledge, for the first time.

## Results and Discussion

### Matrix effects on the acidic properties

The acidic properties of the three individual catalyst components, herein named zeolite (i.e., zeolite Y), binder (i.e., boehmite/silica), and clay (i.e., kaolin clay), and of the three spray‐dried samples, further denoted ZeBi (i.e., zeolite‐binder), BiC (i.e., binder‐clay), and a full‐fetched FCC (i.e., zeolite‐binder‐clay), were analyzed with the temperature‐programmed desorption of NH_3_ (NH_3_‐TPD) and FTIR spectroscopy in combination with CO as a probe molecule for acid sites (CO FTIR).

#### Temperature‐programmed desorption of NH_3_

**Figure 1:**
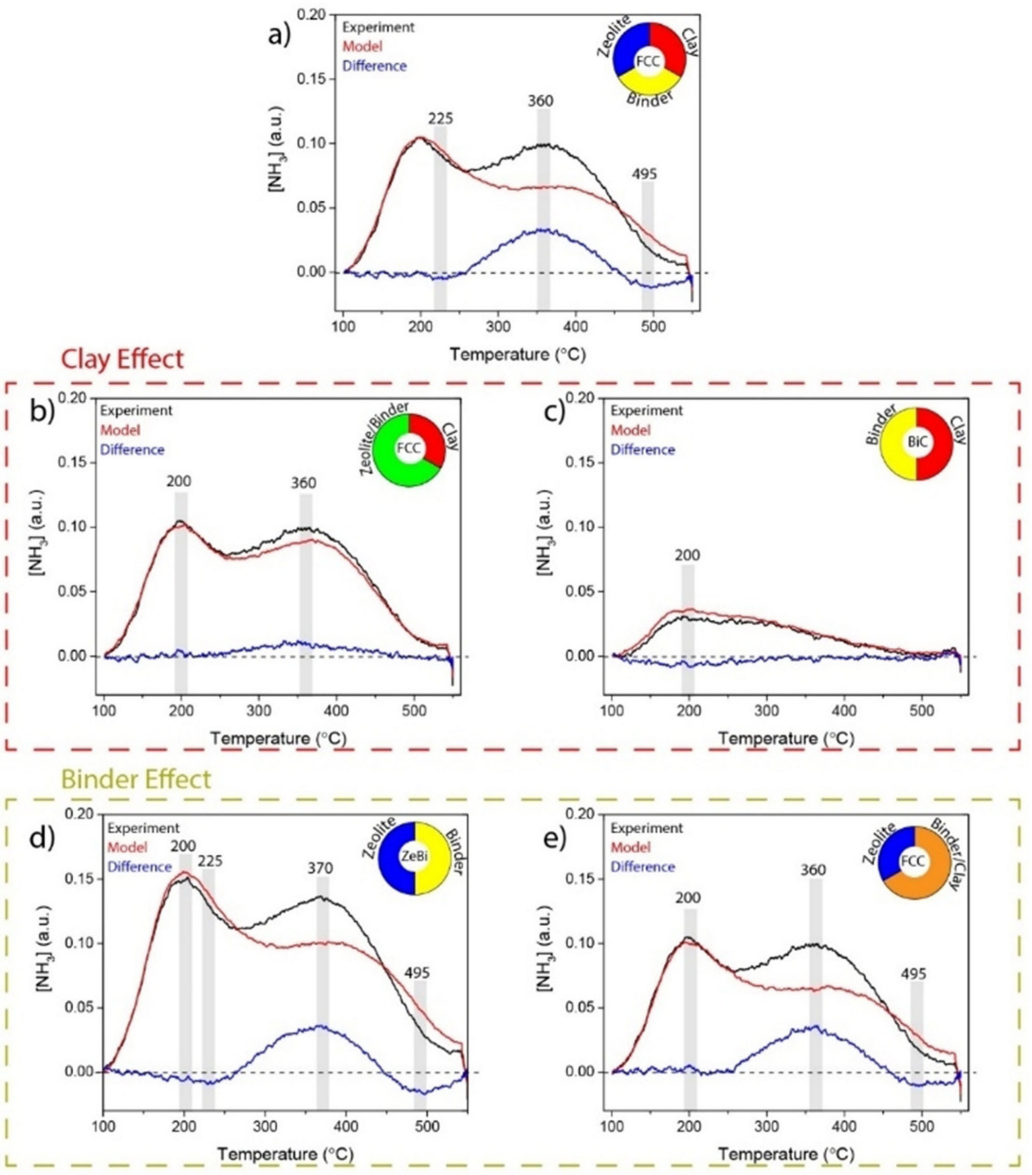
Matrix effects on the acidic properties of the zeolite in fluid catalytic cracking (FCC) catalysts, as measured with temperature programmed desorption (TPD) of NH_3_. The experimental plot (black) is compared to the modeled plot (red) composed of the components indicated in the pie chart. The binder effect is shown in blue. a) FCC composed of its components, b) FCC composed of clay and ZeBi, c) BiC composed of its components, d) ZeBi composed of its components, and e) FCC composed of zeolite and BiC. All curves are corrected for sample weight and the models were based on weight‐corrected plots.

The individual TPD profiles for the six samples under study are shown in Figure S1 in the Supporting Information, while Table 1 summarizes the quantified amount of acid sites (expressed as mmol g^−1^) per sample from the NH_3_‐TPD analysis. This amount includes the whole range of Brønsted and Lewis acid sites with different strengths. The zeolite component is the most acidic sample with ≈1.34 mmol g^−1^ acid sites in comparison to the binder (≈0.38 mmol g^−1^) and clay (≈0.03 mmol g^−1^), as was also evidenced from the NH_3_‐TPD profile intensities in Figure 1. It is interesting to look at the acidic properties of the spray‐dried samples. Upon the interaction between the zeolite and binder component, additional acid sites are created. With a physical 1:1 mixture of the two components, ≈0.86 mmol g^−1^ of acid sites were expected. Instead, the ZeBi sample exhibits an acidity concentration of ≈0.92 mmol g^−1^. This is considered the matrix effect on acidic properties.^7^ Such synergistic effects also take place upon the formation of the FCC sample (≈0.66 mmol g^−1^ in comparison with the expected ≈0.58 mmol g^−1^). The interaction between binder and clay, on the other hand, results in an almost negligible diminishing of acid sites.

**Table 1:** Overview of the accumulated integrated amount of NH_3_ desorbed from the different samples under study as studied with temperature programmed desorption (TPD).

Sample	Amount of acid sites
	[mmol g^−1^]
Zeolite	1.34
Binder	0.38
Clay	0.03
ZeBi	0.92 (0.86)^[a]^
BiC	0.20 (0.21)^[a]^
FCC	0.66 (0.58)^[a]^

[a] This value is the expected value based on a 1:1 physical mixture of the single components

To understand which type of acid sites are affected, created, or removed upon the interaction between different components, we have modeled the NH_3_‐TPD profiles of the spray‐dried samples based on a pure physical 1:1 weight ratio mixture of the single component and plotted them in the same graph with the experimentally obtained NH_3_‐TPD profiles from Figure S1 to observe matrix effects. The results are shown in Figure 1. In each graph, the experimentally obtained NH_3_‐TPD profile is indicated in black, the model in red, and the difference of these two in blue. The difference plot represents the synergistic or antagonistic matrix effect. The pie chart in the top right corner of each graph illustrates which sample was modeled and how the model was built. As such, the matrix effects, as observed for the fully mounted FCC catalyst in Figure 1 a, are now assorted in Figure 1 b–e, where the influence of clay and binder on the acidic properties is demonstrated in detail.

The difference between the experimental and modelled profile for the FCC catalyst relies on the increase of relative weak Brønsted acid sites (≈360 °C)^26^ and the decrease of acid sites at ≈225 °C and ≈495 °C.^7^, ^26^, ^27^ The former is ascribed to weaker Lewis acid sites and the latter corresponds to NH_3_ desorbing from strong Lewis acid sites.^27^ The increase in Brønsted acid sites is mainly caused by the interaction between zeolite and silica/boehmite binder (Figure 1 d) and, to a lesser extent, through the interaction with the kaolin clay (Figure 1 b). Whiting et al. previously reported the influence of a pure alumina binder on the acidic properties of zeolite ZSM‐5, proposing the migration of aluminum into the zeolite framework, creating additional Brønsted acid sites.7a, 9a In this work, prior to the treatment with probe molecules NH_3_ or CO, the samples were dried at 550 °C for 1 h. In correspondence with the work from Whiting and co‐workers, we propose that it is possible that, upon this in situ calcination, while the boehmite in the binder undergoes phase transformation,^28^ a mobile alumina species is formed that is able to migrate into the zeolite framework, thereby creating additional Brønsted acid sites.

The decrease in strong Lewis acid sites (≈495 °C) is caused by an interaction between the zeolite and binder material (Figure 1 d,e), while the clay does not seem to affect the strong Lewis acidic properties in the FCC catalyst (Figures 1 b,c). The matrix effect on the presence of weak Lewis acid sites (≈200 °C) in the FCC catalyst is negligible as a net result of different interactions. When two different components interact, it results in either a small increase or decrease of weak Lewis acid sites. Within the full FCC catalyst, however, these effects have practically cancelled each other out. Coordinatively unsaturated (aluminum) sites (CUS) are considered strong Lewis acid sites.^29^, ^30^, ^31^, ^32^ Upon aqueous mixing with other catalyst components, these sites are the most reactive and are likely to reorganize with other reactive CUS in the mixture. As such, strong Lewis acid sites on the edge of the zeolite domains can interact with the CUS from the binder. As a result, these Al centers saturate their coordination sphere, losing their strong Lewis acidic properties. In other words, the loss of Lewis acid sites is directly related to the interaction between matrix and zeolite within the FCC body.

Overall, the matrix effects are most pronounced upon the interaction between the zeolite and binder components. The net matrix effect as observed for the fully mounted FCC catalyst (Figure 1 a) strongly resembles the binder effect within the ZeBi sample (Figure 1 d). The influence of the clay component on the acidic properties of the FCC catalyst is less pronounced, but not negligible. A small amount of Brønsted acid sites is created due to the interaction between clay and zeolite/binder (Figure 1 b) and the number of Lewis acid sites decreases due to the interaction between clay and binder (Figure 1 c). The loss of Lewis acid sites can also be explained by the decreased materials accessibility.

#### FTIR spectroscopy of CO adsorption

The acidic properties of the FCC catalyst, its individual components, and the two‐component spray‐dried samples were also studied with FTIR spectroscopy making use of CO as a probe molecule. Figures S2 a and S2 b in the Supporting Information summarize the FTIR spectra of the six samples before (black) and after CO adsorption (red) in the OH and CO vibrational region, respectively. In the CO vibrational region (2250–2050 cm^−1^), the blue‐shift of the original CO stretching vibration (i.e., 2143 cm^−1^ in its liquid state) upon interaction with an acid site, can be taken as a measure of the acidic strength and nature. By looking at the corresponding consumption of the bands in the OH vibrational region (3800–3500 cm^−1^), a structure‐acidity correlation can be established.^33^, ^34^, ^35^, ^36^ For a more detailed discussion of the individual spectra, we refer to the Supporting Information. A striking observation from these spectra, however, is that the OH vibrational bands at ≈3695 and ≈3745 cm^−1^, assigned to aluminol and silanol groups, respectively, show a significant blue‐shift upon the interaction of CO with Lewis acid sites. When CO donates electron density into the empty orbital of the Lewis acidic aluminum center, this transfer is possibly stabilized by transferring some of the electron density into the neighboring O−H bond. This strengthens the O−H bond, causing a blue‐shift in the FTIR spectrum. The indirect indications of Lewis acidity via the observed perturbation of hydroxyl groups has been demonstrated before by Busca and co‐workers.^37^ This observation can provide important information on the structural origin of Lewis acid sites in the FCC catalyst, as it indicates that Lewis acidic Al sites in the FCC catalyst are in the near proximity of hydroxyl groups. In particular, Figure 3 demonstrates that the Lewis acid sites near silanol groups (≈3742 cm^−1^) are inherent to the zeolite and binder domains, whereas strong Lewis acid sites near aluminol groups can originate from the clay (3695 and 3675 cm^−1^) or the zeolite (3670 cm^−1^).

**Figure 2:**
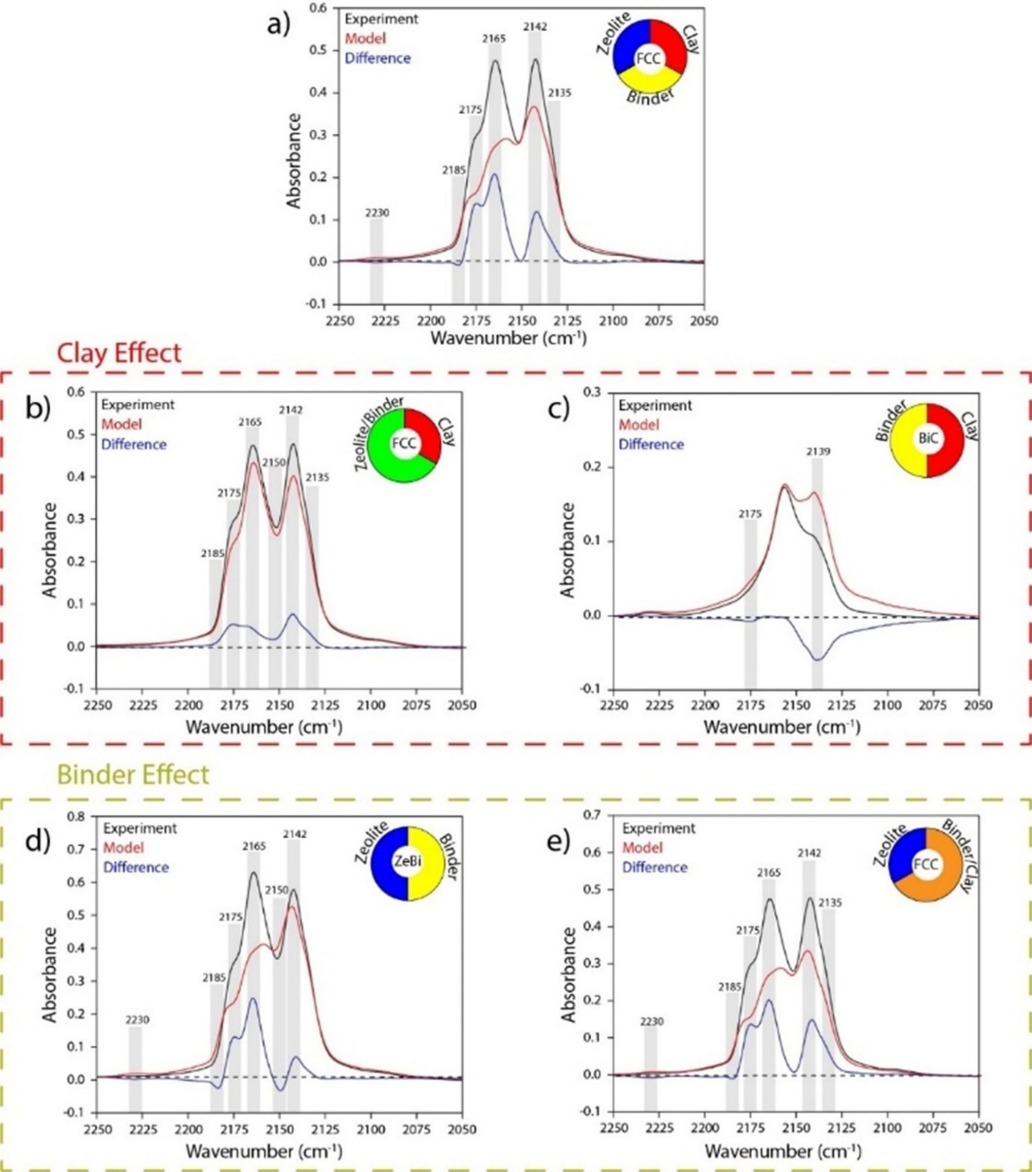
Matrix effects on the acidic properties of the zeolite in fluid catalytic cracking (FCC) catalysts, as revealed by CO FTIR spectroscopy. The experimental spectrum (black) is compared to the modeled spectrum (red) composed of the components indicated in the pie chart. The matrix effect is shown in blue. a) FCC composed of its components, b) FCC composed of clay and ZeBi, c) BiC composed of its components, d) ZeBi composed of its components, and e) FCC composed of zeolite and BiC. All models were based on weight‐corrected spectra.

Matrix effects, as determined through the comparison between experimental and model NH_3_‐TPD profiles in Figure 1, were also established with CO FTIR spectroscopy. Since the FCC catalyst is composed of zeolite, binder, and clay in a 1:1:1 weight ratio, the CO FTIR spectrum of the FCC catalyst can be modeled via a linear combination of the weight‐corrected CO FTIR spectra of the zeolite, binder, and clay, similar to the models used for the NH_3_‐TPD profiles. The result of this linear combination model is included in Figure 2 (red), together with the experimentally obtained spectrum of the FCC catalyst (black). The difference between the model and the experiment is considered to be the matrix effect and is depicted in blue.

The matrix effects, as observed for the fully mounted FCC catalyst in Figure 2 a, are assorted in Figures 2 b–e, where the effects of clay and binder on the acidic properties are demonstrated in detail. It is important to note here, that small differences between the model and the experimental spectrum can also derive from minor inconsistencies in the model, thus to the experimentally assumed equal sample density, neglecting small differences in for example, IR transmission.

Interestingly, in correspondence with the results from NH_3_‐TPD, the zeolite‐binder interactions (Figure 2 d) dominate the matrix effects observed for the FCC catalyst in Figure 2 a. The increase in Brønsted acid sites is mainly caused by the interaction between the binder and zeolite, as observed in Figure 2 d with increasing intensity at ≈2175 and ≈2165 cm^−1^.^37^, ^38^, ^39^ This increase was also observed in the NH_3_‐TPD results in Figure 1. The band at ≈2165 cm^−1^, corresponding to weak Brønsted acid sites increases more strongly in comparison to the band at ≈2175 cm^−1^, assigned to strong Brønsted acid sites. This implies that both types of Brønsted acid sites are formed upon the interaction between binder and zeolite, but the weak ones prevail. This also explains the fact that the average Brønsted acid site weakens in strength, as was observed in the NH_3_‐TPD results in Figure 1.

In accordance with the NH_3_‐TPD results, strong Lewis acid sites (characterized by an absorption at ≈2230 cm^−1^)^30^, ^40^ are suppressed upon the interaction between the zeolite and the binder material, but remain present in the BiC sample (Figure 2 c). This suggests that strong Lewis acid sites in the binder and zeolite are located on the edge of their respective domains, while they are protected inside the layered clay domains. Except for a clear dilution of the acidic properties and the accessibility (decrease of the peak at ≈2139 cm^−1^), the spectral features for the BiC sample simply seem to be the average of the individual two components. Interestingly, the strong Lewis acid sites (at ≈2230 cm^−1^) in the binder are preserved upon interaction with the clay component, whereas these had disappeared upon the interaction between binder and zeolite. This either implies that the clay prevents the full removal of strong Lewis acid sites in the FCC catalyst as a result of zeolite‐binder interactions or it means that the strong Lewis acid sites in the FCC catalyst all originate from the clay.

### Matrix effects on accessibility properties

The synergistic or antagonistic effects on the acidic properties of the FCC catalyst upon individual component interactions can only be probed when the sites are. Also, it is important to note that acid sites are only relevant when reactants can reach these acid sites during the FCC process within the riser reactor. To investigate the accessibility of the six samples under study and assess the extent of matrix effects, Ar physisorption was employed. The corresponding isotherms are depicted in Figure S3 in the Supporting Information and the quantified surface areas and pore volumes are summarized in Table 2. For the spray‐dried samples ZeBi, BiC, and FCC, the expected values for the BET surface area, pore volume, micropore surface area, and micropore volume are also indicated. These values are calculated based on the 1:1 physical mixture of the components without any additional matrix effects. Interestingly, Table 2 demonstrates that the binding of different components together in a spray‐dried particle, can affect the accessibility of the resulting samples.

**Table 2:** Overview of the structural properties, including (micropore) surface area and (micro‐)pore volume of the different samples under study, as determined with Ar physisorption.

Sample	BET surface area	Pore volume	*t‐*Plot micropore surface area	*t‐*Plot micropore volume
	[m^2^ g^−1^]	[cm^3^ g^−1^]	[m^2^ g^−1^]	[cm^3^ g^−1^]
Zeolite	504	0.22	260	0.10
Binder	194	0.13	0	0
Clay	21	0.04	0	0
ZeBi	395 (349)^[a]^	0.19 (0.18)^[a]^	250 (130)^[a]^	0.07 (0.05)^[a]^
BiC	84 (108)^[a]^	0.08 (0.09)^[a]^	0	0 (0)^[a]^
FCC	287 (240)^[a]^	0.15 (0.13)^[a]^	180 (87)^[a]^	0.05 (0.03)^[a]^

[a] Expected value based on the single components.

The zeolite component has the highest surface area with a value of ≈504 m^2^ g^−1^, followed by the binder with a value of ≈194 m^2^ g^−1^. Kaolin is a very dense material with a small surface area (≈21 m^2^ g^−1^) and a negligible pore volume. The binder contains mesopores, while the zeolite contains both micropores and mesopores. Upon the mixing of components, spray‐drying, and subsequent calcination, some interesting matrix effects are observed. The ZeBi sample contains a larger surface area than expected. This is mainly accompanied with a larger micropore volume. This means that the silica/boehmite binder and zeolite component interact together during synthesis conditions and create additional micropores, resulting in a larger surface area. An opposite effect occurs upon the mixing of the binder and clay component.

The resulting BET surface area is lower than expected (≈84 m^2^ g^−1^ in comparison to the expected value of ≈108 m^2^ g^−1^). Also, the total pore volume decreases. We ascribe this to the dense clay component filling and blocking the larger pores and channels. This is in accordance with the observed decrease in physisorbed CO, as evidenced by the 2139 cm^−1^ FTIR peak in Figure 2 c. In the fully shaped FCC material, we observe similar patterns as observed for ZeBi. This indicates that the clay does not interfere with the zeolite‐binder interactions. The increase in surface area due to zeolite‐binder interactions in combination with a decrease in surface area due to binder‐clay interactions, therefore, gives a net result of a surface area increase.

### Matrix effects on structural properties

The acidic and accessibility properties of an FCC catalyst are considerably influenced by matrix effects, as clearly indicated by the results from the NH_3_‐TPD and CO FTIR spectroscopy measurements, pointing towards effects on acidity, as well as by the results from Ar physisorption, pointing towards effects on accessibility. Here, we will discuss the structural nature of interactions between the different components in a FCC catalyst.

**Figure 3:**
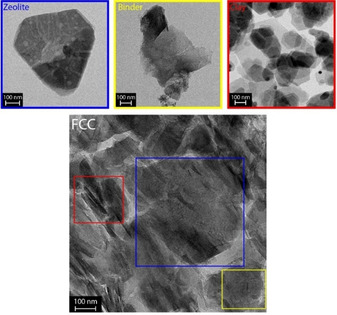
Transmission electron microscopy (TEM) micrographs of the microtomed fluid catalytic cracking (FCC) catalyst and the individual components zeolite (blue), binder (yellow), and clay (red). The presence of the individual components is highlighted in the FCC micrograph using squares in the corresponding colors.

Figure 3 shows a TEM image of a microtomed FCC catalyst sample in which the three different components are highlighted in blue (zeolite), yellow (binder) and red (clay). The recorded TEM images of the three individual FCC catalyst components are also illustrated. The FCC morphology is a clear heterogeneous mixture in which different components can clearly be distinguished. The zeolite consists of large crystalline structures of ≈700 nm with visible mesopores. The clay consists of smaller crystallites ≈200 nm in different shapes, which, when overlaid, are difficult to distinguish as single crystallites. The binder comprises three components with different morphologies, namely the amorphous spherically shaped silica particles mixed with both crystalline and amorphous boehmite.

To further identify the presence of all components in the FCC catalyst and other two spray‐dried samples (i.e., ZeBi and BiC), XRD patterns of all six samples were recorded. The results are summarized in Figure S4 of the Supporting Information. The XRD patterns confirm the presence of crystalline zeolite H‐Y in the zeolite component, the boehmite in the binder, and the kaolinite in the clay component. Furthermore, it can be observed that the method of aqueous mixing and subsequent spray‐drying does not affect the crystallinity of these individual FCC components. Indeed, the FCC catalyst reveals an XRD pattern that preserves all relevant XRD peaks from the individual FCC components, suggesting a physical mixture. Also, the ZeBi sample and BiC sample seem merely physical mixtures of their respective single components based on the XRD patterns.

Whereas structural characterization of the FCC catalyst, the ZeBi and BiC sample, and individual components did not demonstrate a significant interaction between the components, the study into the acidic properties of these samples revealed clear matrix effects. The main differences between the structural and acidity study concerns the sample pre‐treatment. In order to study the acidic nature of a solid surface, samples require a heat‐treatment to remove adsorbed water molecules from the acid sites. At the employed drying temperatures, the samples are subject to structural changes. Matrix effects, as demonstrated in the previous section, can thus be better rationalized with a structural study under similar conditions. To that extent, samples were heat‐treated at 550 °C under a N_2_ flow in a fluidized bed reactor for 1 h prior to XRD, TEM and MQ‐MAS NMR measurements to mimic the in situ sample pre‐treatment in the acidity studies and establish matrix effects in terms of structure‐acidity relations.

#### Effects of heat‐treatment on structural properties

**Figure 4:**
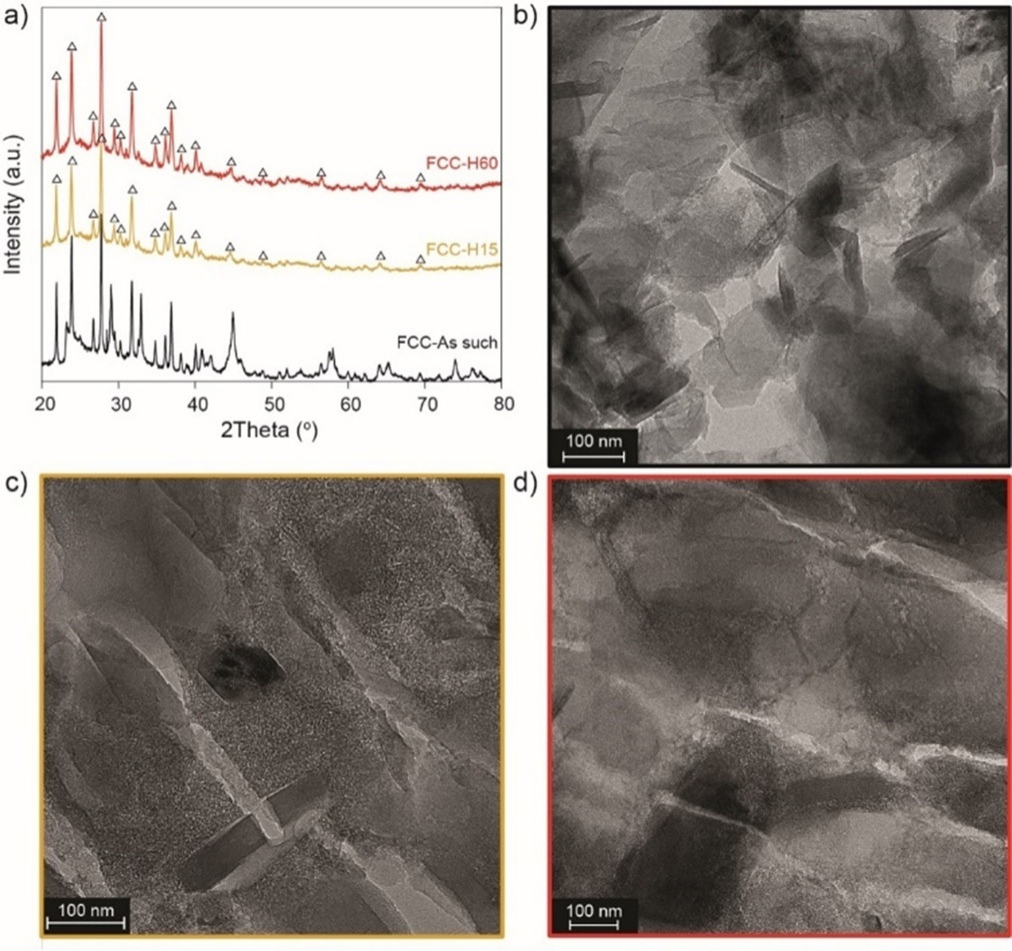
Morphology changes in the fluid catalytic cracking (FCC) catalyst upon prolonged heating, as indicated by X‐ray diffraction (XRD) (a), depicting the patterns for the FCC as made (black), and after 15 min (FCC‐H15, yellow) and 60 min (FCC‐H60, red) of heating at 550 °C under N_2_ flow. The corresponding transmission electron microscopy (TEM) micrographs are shown in b), c) and d), respectively.

Figure 4 a depicts the XRD patterns recorded after 15 (FCC‐H15) and 60 min (FCC‐H60) of heat‐treatment, respectively. Upon a heat‐treatment at 550 °C under N_2_ flow in a fluidized bed reactor, the results are different from what was observed in Figure S4. The XRD patterns are compared with the FCC as such, that is the FCC catalyst prior to heat‐treatment. All XRD peaks corresponding to the crystalline zeolite are indicated with a triangle. Already after 15 min of heat‐treatment, all XRD peaks corresponding to the binder and clay materials have disappeared and only the zeolite material has preserved its crystallinity upon prolonged heating of the FCC catalyst.

Figures 4 b–d depict the TEM images of the microtomed FCC catalysts before and after heat‐treatment and confirm the changing morphology of the FCC catalyst with prolonged heating times. Whereas different FCC components could still be distinguished in the fresh FCC catalyst, the morphology has become more homogeneous and amorphous upon prolonged heating times. This indicates that not only did the components lose their crystallinity, but they have also interacted with each other and merged in a new active matrix phase.

**Figure 5:**
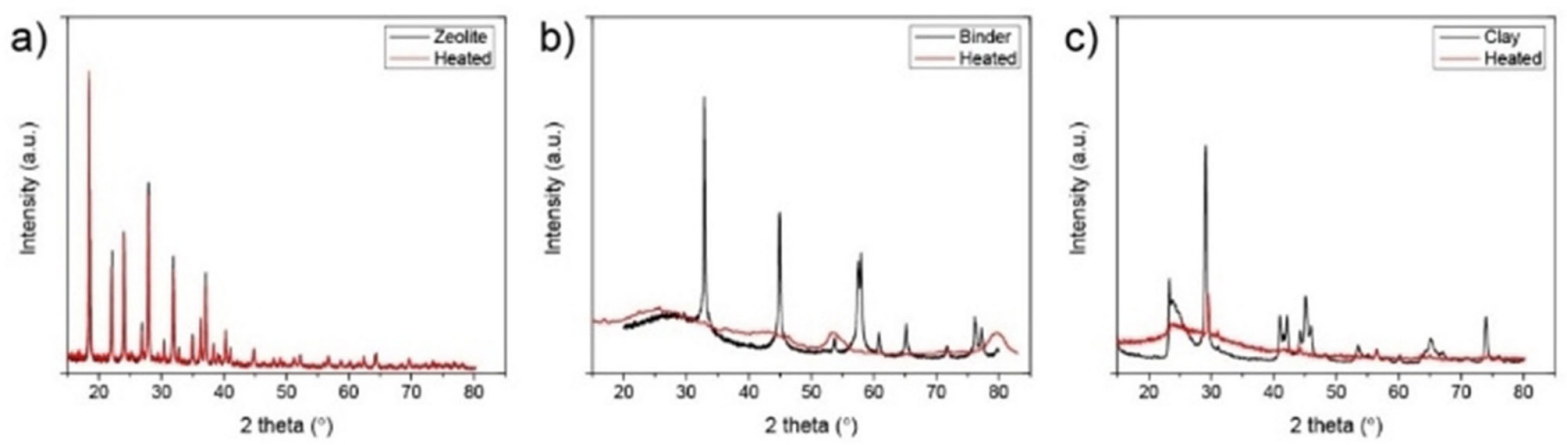
X‐ray diffraction (XRD) patterns of a) zeolite, b) binder, and c) clay. The pattern before heating is depicted in black and the pattern after heat‐treatment is indicated in red.

This is also demonstrated in Figure 5 that presents the XRD patterns of the single components before and after heat‐treatment. The decrease in crystallinity of the binder (Figure 5 b) is associated with the phase transformation of boehmite into γ‐alumina, which has also been reported in literature.^28^, ^41^ The kaolin clay (Figure 5 c) is known to transform into amorphous *meta*‐kaolinite upon heating to 500 °C.^42^ Zeolite H‐Y, on the other hand, is capable of preserving its crystallinity upon heating, as indicated in Figure 5 a.

The six samples under study were further analyzed with ^27^Al MQ‐MAS NMR spectroscopy before and after heat‐treatment to investigate the structural changes that are accompanied with heat‐treatment. Nuclear magnetic resonance (NMR) of quadrupolar nuclei, such as aluminum in zeolites, is a powerful spectroscopic technique.^43^, ^44^, ^45^, ^46^ Due to its ability to provide insights into the local atomic environment of aluminum, it is a valuable method to probe the structure of the materials under study. ^27^Al NMR spectroscopy is particularly useful in the case of zeolite‐type catalysts since it makes it possible to monitor the structural changes that occur in a material when exposed to different treatments.^25^, ^43^, ^47^, ^48^, ^49^, ^50^, ^51^, ^52^, ^53^, ^54^, ^55^, ^56^, ^57^, ^58^ ^27^Al multiple quantum (MQ) MAS NMR is especially useful for the study of disordered materials, allowing the acquisition of well‐resolved spectra that have an isotropic dimension, free of any anisotropic quadrupolar broadening.^47^, ^58^, ^59^, ^60^, ^61^, ^62^, ^63^ This provides good resolution for the various Al coordination environments in the sample, giving insights into the distribution in the NMR parameters and thereby allowing a more detailed characterization of such materials. Based on the chemical shift, three regions can be distinguished in ^27^Al MQ‐MAS NMR spectra: 0–20 ppm, 30–50 ppm and 50–80 ppm, which are ascribed to octahedral, penta‐coordinated and tetrahedral aluminum coordination environments, respectively, as is often assigned in the literature.^47^, ^51^, ^57^, ^58^, ^60^, ^61^, ^64^, ^65^

**Figure 6:**
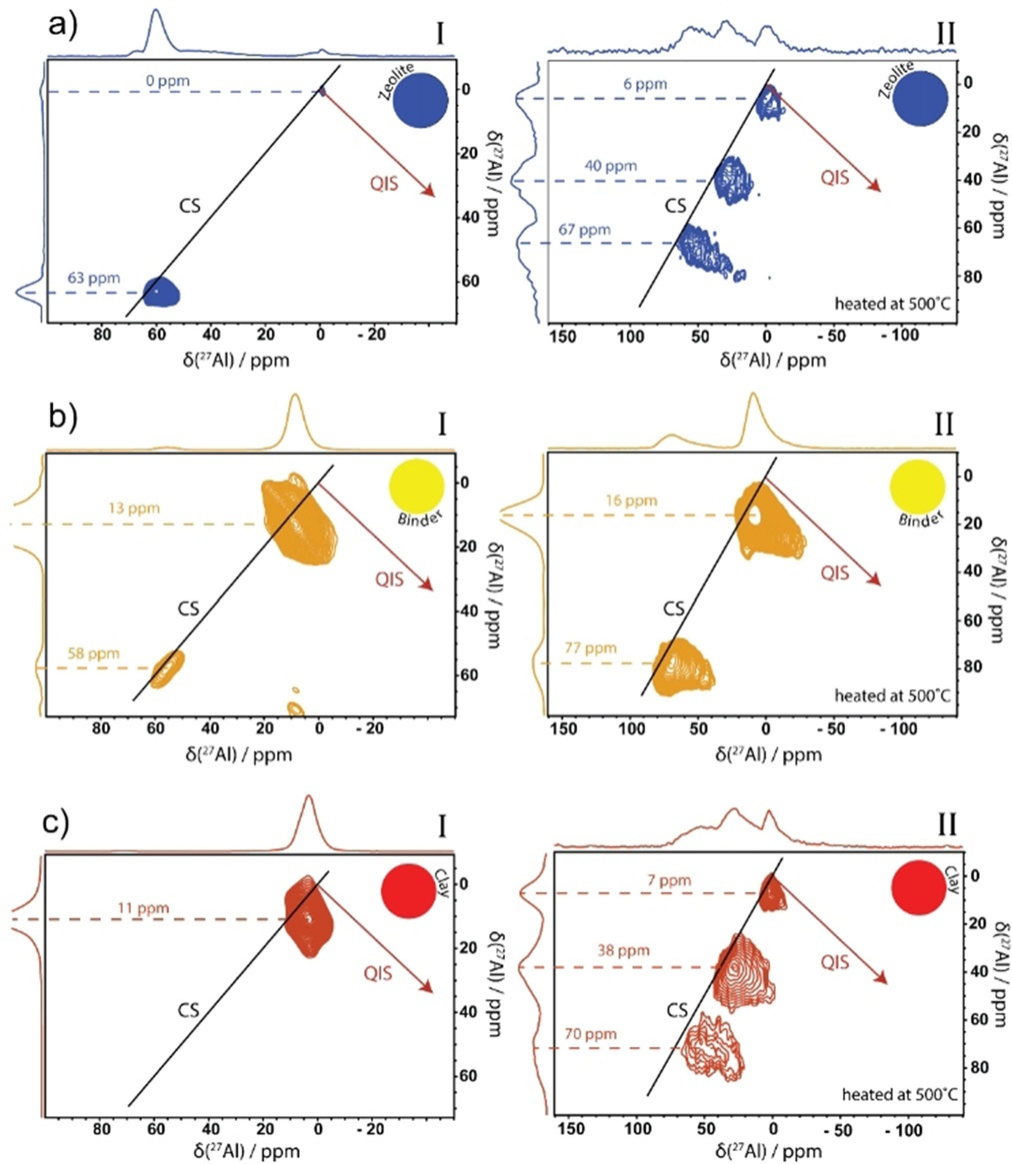
^27^Al 3Q magic angle spinning (MAS) nuclear magnetic resonance (NMR) spectra of the single components (a) zeolite, b) binder, c) clay) before (panel I) and after (panel II) the heat‐treatment with the projections along the F1 (vertical) and F2 (horizontal) axis.

Figure 6 demonstrates the ^27^Al 3Q‐MAS spectra for the three components before (panel I) and after heat treatment (panel II). Looking at the untreated samples in panel I, the zeolite mainly contains tetrahedral aluminum (≈63 ppm) in the zeolite Y framework, which is the origin of Brønsted acid sites.^6^ The binder contains silica and boehmite species, demonstrating mainly octahedral aluminum (≈13 ppm) with a small amount of tetrahedral aluminum (≈58 ppm). Since pure boehmite is known to have merely octahedral aluminum centers,^66^ these tetrahedral aluminum species must be the result from an interaction between boehmite and silica on the interface, creating tetrahedral Al centers in a silica environment. The clay component is kaolin, which is a layered mineral, connecting tetrahedral silica sheets with octahedral alumina sheets.^67^, ^68^ This is confirmed in the ^27^Al MQ‐MAS NMR spectra, shown in Figure 6 c, with one large peak for octahedral aluminum species.

The smaller chemical shift for the Al centers in the binder (≈58 ppm) in comparison to the zeolite Al centers (≈63 ppm) indicates a higher level of electron shielding from the Al nucleus, due to a slightly larger coordination number on average.^47^ Furthermore, the tetrahedral peak of the binder material is more elongated along the “chemical shift (CS) axis”, indicating a higher level of disorder in the binder material than in the zeolite material with a resulting wider distribution in isotropic chemical shifts.^60^ This indicates that the tetrahedral Al species in the zeolite and binder materials have different conformations. The zeolite peak, on the other hand, demonstrates a larger distortion along the F2 axis caused by an asymmetric charge distribution, that originates from the corresponding Brønsted acidic proton.^47^ The octahedral Al peak in the binder also demonstrates a small elongation along the CS axis, but a bigger quadrupolar interaction can immediately be recognized from the increased line‐broadening.^61^ This indicates similar octahedral Al conformations with reduced symmetry of environment, resulting in the increase of the electric field gradient and, therefore, of the quadrupolar product.^65^

Panel II in Figure 6 presents the ^27^Al MQ‐MAS NMR spectra after heat‐treatment. The heat‐treatment of the samples results in the loss of physisorbed water and chemisorbed water from the Brønsted and Lewis acidic sites. This is witnessed with a strong increase in the average quadrupole coupling constant and in the irregular shape of all peaks in the MQ‐MAS NMR spectra of the heat‐treated samples. Indeed, dehydration of the sample is accompanied by a decrease in the symmetry around the Al atoms and, thus, by an increase of the NMR linewidths.^65^ Moreover, we recognize higher chemical shifts for all NMR peaks. This indicates that, overall, the Al centers experience a decreased electron density in the first coordination sphere. It is proposed that upon dehydration, the oxygen atoms around Al become more polarized. This leads to increased levels of electron density deshielding of the Al atoms, causing the increasing chemical shift.

In the zeolite material, we notice an increment of signal corresponding to octahedral and penta‐coordinated Al and an elongation of the tetrahedral Al peak along the quadrupole induced shift (QIS) axis. This suggests the partial dealumination of the zeolite framework and consequent transformation of framework Al to extra‐framework Al species and demonstrates the reactivity of these tetrahedral Al centers at elevated temperatures.^58^ The tetrahedral Al sites are isolated in a tetrahedral silica framework and are the origin of Brønsted acid sites. These sites are surrounded with water at room temperature. During heat‐treatment, these sites are dehydrated, inducing much more strain in the network because of the electrostatic forces of dehydrated acidic protons that tend to affect the local environment. The resulting asymmetric environment of the Al atoms, therefore, leads to much larger electric field gradients.

The deshielding effect is minimum for the tetrahedral Al sites (≈63 to ≈67 ppm) but more evident for the octahedral Al peak, as opposed to the binder, where the deshielding is stronger for the tetrahedral Al site, with a significant downfield shift of more than 10 ppm. This is ascribed to the phase transformation of boehmite into alumina upon heating. The appearance of the 77 ppm tetrahedral Al peak is, therefore, at the expense of the octahedral boehmite peak at ≈13 ppm and corresponds to an alumina phase. Since the binder demonstrates Brønsted acidic properties, the tetrahedral Al at ≈58 ppm is probably still present but is hidden under the larger ≈77 ppm peak. This peak corresponds to tetrahedral Al in a silica environment. The Al coordination of the clay is only octahedral but upon heat‐treatment, the appearance of tetrahedral and especially pentahedral sites is observed. Furthermore, interestingly, the octahedral resonance shifts upfield after heating, indicating the presence of a higher electron density, possibly the result of a different geometry.

**Figure 7:**
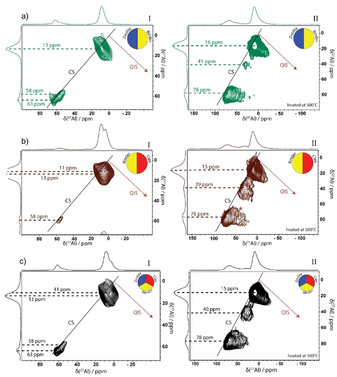
^27^Al 3Q magic angle spinning (MAS) nuclear magnetic resonance (NMR) spectra of the spray‐dried samples ZeBi (a), BiC (b), and FCC (c) before (panel I) and after (panel II) the heat‐treatment with the projections along the F1 (vertical) and F2 horizontal) axis.

Figure 6 demonstrated clearly which Al centers in the individual components become reactive upon a heat‐treatment. A reaction between these centers could lead to the matrix effects, as observed in Figures 1 and 2. Figure 7 presents the ^27^Al‐sheared 3Q‐MAS NMR spectra of the three spray‐dried samples before and after heat‐treatment. The spectra in Panel I show the different Al coordination environments present in the spray‐dried samples before heating. Within the FCC catalyst (Figure 7 c), mainly two types of Al species are observed. The small peak at ≈60 ppm corresponds to tetrahedral Al species and the larger peak at ≈12 ppm corresponds to octahedral Al.^57^ Closer inspection of this spectrum reveals that both peaks actually comprise two separate peaks. These individual peaks can be traced back to one of the single components, as observed in Figure 6. The tetrahedral Al peaks in the FCC catalyst originate from the zeolite (≈63 ppm) and the binder (≈58 ppm). The octahedral Al peak also consists of two individual contributions. Both the binder (≈13 ppm) and the clay (≈11 ppm) contribute to the octahedral Al species in the FCC catalyst material.

The ZeBi sample shows a strong octahedral Al contribution at ≈13 ppm with similar spectral features and broadening as observed for the binder component. The tetrahedral region comprises two separate peaks at ≈58 and ≈63 ppm, assigned to the binder and zeolite material, respectively. The BiC sample also seems a merely physical mix of the binder and clay component. The Al species mainly adopt an octahedral coordination with two distinct peaks at ≈11 and ≈13 ppm, assigned to the clay and binder, respectively. The small tetrahedral contribution at ≈58 ppm originates from the binder component. Interestingly, the tetrahedral Al peak seems slightly less distorted for the FCC catalyst than for the ZeBi sample. This indicates that the addition of clay to the spray‐dried catalyst results in the stabilization of the tetrahedral Al species with less induced strain in the particle.

Panel II demonstrates the influence of a heat‐treatment on the structural properties of the spray‐dried samples. Interestingly, whereas the spray‐dried samples appeared merely physical mixtures of the individual components, based on the MQ‐MAS NMR spectra, this is not the case for the samples after heat treatment. This is in correspondence with the XRD and TEM results before and after heat‐treatment and confirms that matrix effects come into play at elevated temperatures.

For all three samples, line broadening patterns are similar to what was observed for the individual binder component. This suggests that the NMR resonances in the FCC, ZeBi, and BiC samples are dominated by the binder signal. The fact that the individual binder component also showed the highest signal‐to‐noise ratio supports this hypothesis, since it indicates that most Al centers originate from the binder component. The downfield shift of the tetrahedral Al species that occurs in all three samples, is ascribed to the phase transformation of boehmite in the binder to alumina upon heating. Upon heating, we observe that the tetrahedral Al peaks in Panel II of Figure 7 become strongly dispersed parallel to the F2 axis, meaning that its line width is mainly caused by second order quadrupole broadening, similar as was observed for the binder component upon heat‐treatment. The tetrahedral Al species from the zeolite material cannot be unambiguously observed in Figure 7 a,c, but are probably hidden under the large peak from the binder. This is rationalized by the fact that isolated tetrahedral Al sites in a zeolite framework are the origin of Brønsted acid sites, which are clearly present in the FCC and ZeBi samples, as indicated in Figure S2 of the Supporting Information. The observed high quadrupolar coupling for the zeolite in Figure 6 a, is not as evident in the ZeBi and FCC sample.

In contrast, for all octahedral peaks in Panel II of Figure 7, the distribution in the electric field gradient dominates, observing a strong broadening along the QIS axis of the two‐dimensional spectrum upon heat‐treatment.^61^ This indicates the presence of many different Al species with all slightly different quadrupolar coupling constants, which can be ascribed to the formation of an amorphous matrix. A new pentahedral Al peak becomes evident after heat‐treatment at 550 °C, which can be either ascribed to the dehydration of octahedral Al atoms inherent to the clay or originates from reactive tetrahedral Al in the zeolite that has increased its coordination number after interaction with the binder.

Finally, Figure S5 of the Supporting Information shows the FTIR spectra of the six samples under study after different dehydration conditions and demonstrates that the zeolite and binder are already completely dehydrated when the desorption temperature reaches 550 °C. Clay, on the other hand, requires a full hour of heat‐treatment under vacuum to dry, which is ascribed to the dense structure of the kaolin clay. This means that the surface groups of zeolite and binder become reactive an hour before the clay is capable of interacting with other components. As mentioned before, the reactivity of the components is ascribed to coordinatively unsaturated surface species. At room temperature, these surface groups are saturated with water molecules. Therefore, matrix effects in terms of structure‐acidity are mostly ascribed to the zeolite‐binder interaction, whereas clay plays a dilution and pore‐blocking role. It is possible that—in an industrial environment—with prolonged times in the FCC reactor, the clay will become more active and play a more important role in the catalytic cracking mechanism.

### Structure‐Acidity Relationships

From the characterization of the six samples under study, structure‐acidity relationships can be derived that aid the understanding of matrix effects in FCC catalysts. It is well reported that the origin of Brønsted acid sites are tetrahedral Al centers in a tetrahedral SiO_2_ framework, the most famous example being zeolites.^6^, ^11^ ^27^Al MQ‐MAS NMR spectroscopy in Figure 6 indicates that these Brønsted acid sites are characterized by a peak at ≈60 ppm. The zeolite possessed tetrahedral Al species with an NMR signal at ≈63 ppm, while upon dehydration, there is a small downfield shift to ≈67 ppm since the oxygen atoms around Al become more polarized. The binder also contains Brønsted acid sites, as characterized by an NMR peak at ≈58 ppm.

The interaction between binder and zeolite materials resulted in the formation of additional Brønsted acid sites. Figure 6 a suggests that at elevated temperatures, the zeolite framework can partially collapse due to dealumination, leaving defects in the zeolite framework. In the presence of the binder material, on the other hand, we propose that mobile Al species, originating from the boehmite binder, can insert into these zeolite framework defects, thereby creating additional Brønsted acid sites. This hypothesis on the matrix effects is supported by the Ar physisorption results showing an increase in micropores upon the interaction between zeolite and binder.

The extent of elongation along the QIS axis upon dehydration seems to be correlated with the strength of the Brønsted acid sites.^47^ The average Brønsted acidic strength is high for the zeolite that demonstrates a significant elongation along the QIS axis, whereas this elongation is smaller for the ZeBi and FCC materials. These latter samples indeed contain on average weaker Brønsted acid sites. A possible rationalization for this correlation is the fact that a strong dehydrated acidic proton induces much more strain in the network because of the electrostatic forces than a weak Brønsted acidic proton does, leading to a higher quadrupolar coupling. Further research, however, must be performed to confirm this hypothesis.

In addition to the Brønsted acid sites, the zeolite contains a considerable amount of Lewis acid sites. Since the zeolite component mainly consists of tetrahedral Al centers, this indicates that not all tetrahedral Al is Brønsted acidic. Indeed, part of the tetrahedral Al sites is probed as Lewis acid sites. The postulation that strong Lewis acid sites originate from (distorted) tetrahedral Al sites is supported by the observation that also the binder contains strong Lewis acid sites and a considerable amount of tetrahedral Al sites. Also the clay, after activation at 550 °C for 1 h, demonstrates the presence of tetrahedral Al species and Lewis acid sites. Moreover, it is reported in literature that Al centers can expand their coordination number up to 6 ligands.^37^ Extrapolating this theory, octahedral Al centers should not be acidic as their coordination sphere is saturated, penta‐coordinated Al centers are weak Lewis acid sites, and tetrahedral Al centers could be strong Lewis acid sites. Lewis acidity in zeolites is often ascribed to extra‐framework structures, such as Al(OH)^2+^, Al(OH)_2_^+^, Al(OH)_3_, and AlO(OH), although the exact structures are still debated.^15^, ^69^ In these structures, Al is a trivalent cation, which is highly acidic. The presence of strong Lewis acid sites is, therefore, also often observed in combination with more extra‐framework Al.^15^, ^69^ Literature has also often stated that trivalent Al centers in extra‐framework species re‐coordinate to form extra‐framework clusters.^15^, ^69^ In these clusters, the Al centers can be four up to six‐coordinated and will be detected as such with methodology such as ^27^Al MQ‐MAS NMR spectroscopy.^15^, ^56^ Upon the entrance of a strong basic probe molecule, the Al center disconnects from the cluster and coordinates to the probe molecule. As such, it is characterized as a strong Lewis acid site. It is postulated, in accordance with the hypothesis in this research work, that a strong Lewis acid site is a trivalent Al center that disconnects from a tetrahedral extra‐framework and that a weak Lewis acid site is a tetrahedral or penta‐coordinated Al center that disconnects from an octahedral extra‐framework Al.

The strength of Lewis acid sites does not seem to merely depend on the coordination number. For every sample under study, strong Lewis acid sites always appear to be in the proximity of (non‐acidic) hydroxyl groups, as indicated in Figure S2 c in the Supporting Information. The acceptance of an electron pair by a tetrahedral Al center (strong Lewis acid site) is facilitated by the partial transfer of electron density to nearby hydroxyl groups. Consequently, the O−H bond is strengthened and the Al center expands its coordination number. Without the option to stabilize the received electron density, however, the Al center is only weakly Lewis acidic. This appears to be the case in the ZeBi sample. Although there are many tetrahedral Al sites present in the ZeBi sample, they are either Brønsted acidic (≈60 ppm) or weakly Lewis acidic (≈77 ppm). There is no indication of O−H bond strengthening in Figure S2 that suggests the presence of strong Lewis acid sites. The proximity of (non‐acidic) hydroxyl groups to Lewis acid sites has been observed before and is built on this academic literature.^37^, ^70^, ^71^, ^72^, ^73^ In particular, Lewis acidic extra‐framework structures, such as Al(OH)^2+^, Al(OH)_2_^+^, Al(OH)_3_, and AlO(OH), are all Lewis acidic Al centers in the proximity of hydroxyl groups.^15^, ^69^

Figure 2 d shows that Brønsted acid sites are created upon binder‐zeolite interactions at the expense of strong Lewis acid sites. This suggests that tetrahedral Al centers near the edge of the zeolite domain are often strong Lewis acid sites. Upon heat‐treatment, these centers are extracted from the zeolite framework and replaced with Al from the binder material. Binder‐zeolite interactions lead to the dehydroxylation of the nearby hydroxyl groups. As such, these renewed Al centers are no longer strong Lewis acid sites, but new Brønsted acid sites. The remaining strong Lewis acid sites in the FCC catalyst are inherent to the clay. These sites are protected from interaction as they are hidden in the poorly accessible clay, which is only dehydrated after a full hour of heat‐treatment.

## Conclusions

This work has evaluated the matrix effects in a fluid catalytic cracking (FCC) catalyst in terms of structure, accessibility, and acidity. An extensive characterization study into the structural and acidic properties of the FCC catalyst, its individual components, and samples containing only two components (zeolite‐binder and binder‐clay) was performed at relevant conditions. This allowed drawing conclusions about the role of each individual component, describing their mutual physicochemical interactions, establishing structure‐acidity relationships, and determining matrix effects in FCC catalyst materials.

**Figure 8:**
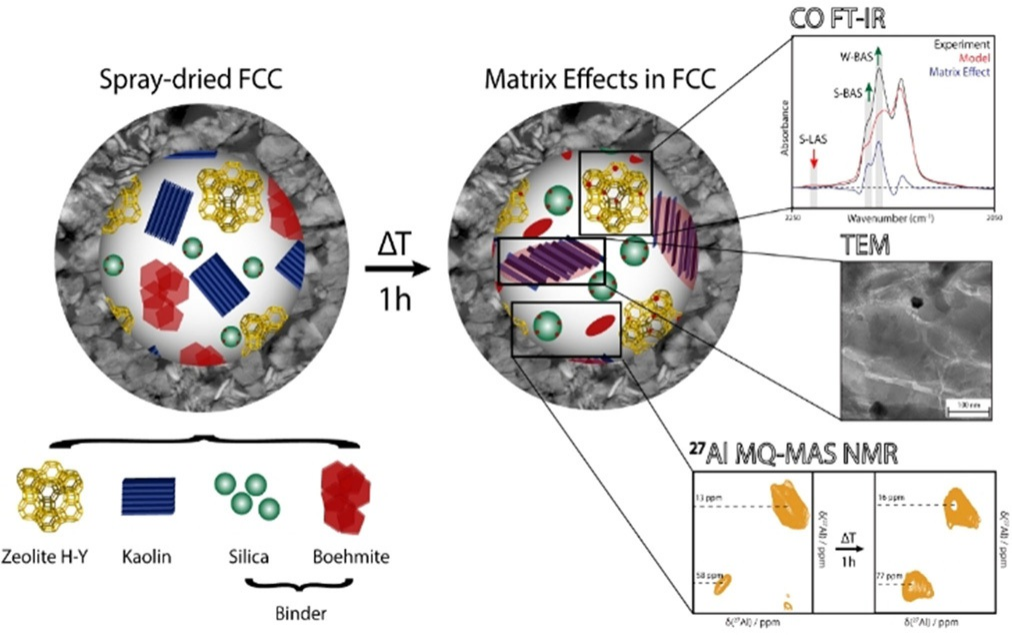
Survey of the experimentally observed matrix effects in fluid catalytic cracking (FCC) catalysts: The spray‐dried FCC catalyst particle consisting of a zeolite H‐Y, kaolin clay, and a silica/boehmite binder, is mainly a physical mixture of its individual components. Only within the binder, the boehmite and silica demonstrate some interaction as evidenced by the peak at 58 ppm assigned to tetrahedral Al centers, responsible for Brønsted acidity in the binder. Upon heat‐treatment for 1 h, however, significant matrix effects come into play. The schematic indicates the matrix effects, as revealed by the used characterization toolbox. FTIR spectroscopy of adsorbed CO on the ZeBi sample indicates the removal of strong Lewis acid sites and creation of Brønsted acid sites. It is proposed to be the result of Al insertion from the binder into the zeolite framework. Transmission electron microscopy (TEM) and X‐ray diffraction (XRD), in combination with Ar physisorption, revealed that the binder and clay lose their crystallinity upon heat‐treatment, and together form an amorphous matrix with reduced accessibility. This is schematically depicted as a red oval (amorphous alumina, instead of crystalline boehmite) that overlays with the partially deconstructed kaolin. Furthermore, advanced nuclear magnetic resonance (NMR) spectroscopy shows the phase transformation of boehmite into alumina in the binder material.

The most important matrix effects are schematically illustrated in Figure 8. It is important to note that the observed matrix effects in this work rely heavily on the structural and acidic properties of the individual FCC catalyst components. This means that, for instance, the use of a different alumina source in the binder or a different zeolite material (e.g., zeolite ZSM‐5 replacing zeolite Y) can lead to different mutual interactions and different acidic properties of the FCC catalyst. The preparation of a spray‐dried FCC catalyst consisting of zeolite H‐Y, kaolin clay, and a silica/boehmite binder results in a practically physical mixture of the individual FCC components. This was demonstrated with TEM, XRD, and ^27^Al MQ‐MAS NMR analysis. The only observed effect in the spray‐dried catalyst was the interaction between silica and boehmite. ^27^Al MQ‐MAS NMR spectroscopy demonstrated a peak at 58 ppm, assigned to tetrahedral Al in a SiO_2_ environment.

Each individual FCC component contributes to a certain extent to the acidic properties of the fully shaped FCC catalyst particle. The zeolite contains the highest amount of Brønsted and Lewis acid sites. These sites originate from tetrahedral Al centers in the tetrahedral zeolite framework. The silica/boehmite binder mainly contains octahedral Al and a small amount of tetrahedral Al in a silica environment, providing minimal Brønsted acidity and strong Lewis acid sites. The boehmite in the binder material is converted into alumina at elevated temperatures that introduces weak Lewis acidity in the FCC matrix. The clay material appears inert, as it preserves a lot of water inside its small pores at high temperatures that blocks acid sites. It, therefore, mainly fills the large pores and blocks a part of the accessibility to the acid sites, hereby fulfilling its role as a diluent. After longer heating times, however, the octahedral Al sites in the clay mineral convert into tetrahedral and penta‐coordinated Al, thereby creating Brønsted and Lewis acid sites.

Upon temperature treatment, however, significant matrix effects come into play. First, an interaction between binder and zeolite occurs that relies on highly reactive Lewis acid sites on the binder‐zeolite interface. We have observed the disappearance of strong Lewis acid sites and the creation of new Brønsted acid sites as a result of this interaction. This was ascribed to mobile Al species inherent to the binder material that are inserted into zeolite defects, most probably at the outer layers of the zeolite particles. Secondly, the interaction between zeolite and binder also results in a significant increase of the micropore fraction. This supports the hypothesis that mobile Al species from the binder insert in zeolite defects, restoring the zeolite framework. Thirdly, the interaction between binder and clay leads to a significant reduction of accessibility, as they merge together forming an amorphous matrix consisting of alumina, silica, and metakaolin. The clay is definitely not inert, as it contains some strong Lewis acid sites that are preserved after 1 h of heat‐treatment, but it does not chemically interact with other FCC components. Therefore, the clay does not play a role in the acidic matrix effects, which are dominated by the zeolite‐binder interaction.

We have demonstrated in this work that even a short exposure of the spray‐dried samples to typical riser temperatures already induces significant modifications in their properties. It must be noted that the state and properties of FCC catalysts, after multiple reaction‐regeneration cycles might be considerably different compared to this 1 h heat treatment. Sintering, coking, metal deposition, ageing etc. will certainly induce severe modifications in the catalyst.

Finally, we were able to correlate structural characteristics to acidic properties in the samples under study. A Brønsted acid site is characterized by a peak around 60 ppm in the ^27^Al MQ‐MAS NMR spectra, corresponding to a tetrahedral Al site in a tetrahedral SiO_2_ environment. The strength of the Brønsted acid site is signified by the extent of elongation along the QIS axis upon dehydration, as this is a measure of the strain in the zeolite framework because of the electrostatic forces caused by an acidic proton. A Lewis acid site is an Al center that can still accept electron density, thereby expanding its coordination number. Based on the described results, it is postulated that the strength of the Lewis acid site depends on two factors. The first parameter is the coordination number, indicated by the downfield shift in ^27^Al MQ‐MAS NMR data. An Al center can coordinate up to six ligands. Therefore, a lower coordination number generally indicates a higher Lewis acidic strength. The second factor involves the facilitation of electron pair acceptance. If an Al center is in close proximity to a hydroxyl group, it can partially transfer the accepted electron density to this hydroxyl group upon coordinating to an additional ligand. These hydroxyl groups can be detected with FT‐IR spectroscopy.

## Experimental Section

### Materials

The spray‐dried fluid catalytic cracking catalyst (further denoted as FCC), the corresponding individual components (further denoted as zeolite, binder, and clay), and the spray‐dried samples containing only two components, namely zeolite and binder (further denoted as ZeBi) and binder and clay (further denoted as BiC) were provided by Albemarle Corporation. The FCC catalyst contains the three individual components (i.e., zeolite H‐Y, binder (silica/boehmite binder) and kaolin clay) in a 1:1:1 weight ratio. The combined spray‐dried model samples ZeBi and BiC contain the two individual components in a 1:1 weight ratio.

The six catalyst samples were used as received for X‐ray diffraction (XRD), transmission electron microscopy (TEM) and multiple quantum magic angle spinning nuclear magnetic resonance (MQ‐MAS‐NMR) measurements. Next, all samples were subjected to a heat‐treatment at approximately 550 °C under a continuous N_2_ flow of 100 mL min^−1^ in a quartz calcination tube. The samples were, subsequently, transferred to a N_2_ glovebox, to prevent air or moisture exposure after the heat‐treatment. Sample preparation for Ar physisorption and MQ‐MAS‐NMR measurements of the heat‐treated samples took place inside the glovebox and samples were transferred under inert atmosphere to the corresponding setups.

### Characterization

Fourier transform infrared (FTIR) spectra were recorded in transmission mode on a PerkinElmer 2000 instrument, equipped with a DTGS detector, using 32 scans per spectrum and a resolution of 4 cm^−1^. Sample preparation took place by pressing approximately 15 mg into a self‐supported wafer that was subsequently placed in a well‐sealed cell with CaF_2_ windows that allows switching between vacuum and the probe molecule gas. Samples were dried at 550 °C (ramp of 10 °C min^−1^) under high dynamic vacuum and kept at that temperature for 1 h. CO (10 % in He, Linde Gas Group, purity 99.9 %) was dosed at low temperatures (−188 °C) and at low pressures with spectra being taken after each pulse. CO desorption occurred through vacuum desorption.

NH_3_ temperature programmed desorption (TPD) experiments were performed on a Micromeritics ASAP2920 apparatus equipped with a TCD detector. Typically, 0.1 g of sample was dried in situ in a He flow with a temperature ramp of 10 °C min^−1^ up to 550 °C and remained at that temperature for 30 min. Subsequently, the sample was cooled to 100 °C; at this point, NH_3_ pulses of 25.17 cm^3^ min^−1^ were applied. After saturation of the acid sites with NH_3_, the sample was outgassed for 2 h at 100 °C to ensure the removal of physisorbed NH_3_. The sample was then heated to 550 °C with a ramp of 5 °C min^−1^ to induce desorption of NH_3_.

X‐ray diffraction (XRD) patterns were recorded on a Bruker AXS Advance D8 apparatus, equipped with Co_Kα_ radiation, operating at 45 kV and 30 mV. The XRD patterns were collected between 20–80°. The samples were prepared outside of the diffractometer. It was assumed that the crystallinity of the heat‐treated samples would not change upon exposure to air.

Transmission electron microscopy (TEM) measurements were performed on a FEI TalosTM F200X instrument. The FCC particles were embedded in an Epofix embedding resin prior to sectioning on a Reichert Jung UltraCut E microtome to 70 nm sections that were placed on 200 mesh copper grids with carbon coated Pioloform film. The individual components were directly placed on the grids.

Ar physisorption was performed at −196 °C using a Micromeritics TriStar instrument. The mesopore volumes (the 2–300 nm range) and Barrett—Joyner–Halenda (BJH) pore size distributions of the silica support and solid activators were determined using the adsorption branch of the isotherm with Aerosil 380 as a reference. Samples were heat‐treated as described above and prepared in the N_2_ glovebox prior to transfer to the Ar physisorption set‐up.

Magic angle spinning nuclear magnetic resonance (MQ MAS NMR) experiments were performed at 11.7 T on a Bruker Avance III spectrometer equipped with a 3.2 mm MAS probe. Spectra were recorded at ambient temperature using 18 kHz MAS. A radio frequency (RF) field of 50 kHz was used for the ^27^Al π/12 pulse followed by 6.5 ms acquisition. 128 scans were accumulated using an inter‐scan delay of 1 s. The ^27^Al chemical shift was externally referenced to an aluminum nitrate solution (Al(NO_3_)_3_(aq)) in milliQ water. The 1D spectra were processed using a line‐broadening of 100 Hz. A zero‐quantum (ZQ) filtered multiple‐quantum magic angle spinning (MQ‐MAS) pulse‐sequence^59^ was used to correlate the ^27^Al isotropic chemical shift (F1) with the quadrupolar line‐shape (F2), specifically the 3Q‐MAS sequence. The RF field for the 3Q excitation pulse was 50 kHz, instead for the soft, selective pulse 3.5 kHz was used. A recycle delay of 1 s and acquisition times of 6.5 ms was used for the direct dimension. For the non‐heated samples an acquisition time of 1.7 ms was used in the indirect dimension, the MQ‐MAS‐NMR spectra were recorded using 948 scans and spectral processing was performed using 100 Hz line broadening in both ^27^Al dimensions. For the heat‐treated samples an acquisition time of 0.9 ms was used in the indirect dimension, the MQ‐MAS‐NMR data were recorded using 1440 scans and spectral processing was performed using 250 Hz line broadening in both ^27^Al dimensions. MQ‐MAS‐NMR data were Fourier transformed and sheared using the software of Bruker Topspin 3.5. Heat‐treated samples were prepared in a N_2_ glovebox. After heat‐treatment, samples were transferred into 3.2 mm rotors inside a N_2_ glovebox, closed with an airtight Teflon cap, and, subsequently, transported under N_2_ atmosphere to the NMR spectrometer. A ^1^H‐NMR spectrum was recorded before and after the experiment to ensure the rotor was airtight.

## Conflict of interest

The authors declare no conflict of interest.

## Supporting information

As a service to our authors and readers, this journal provides supporting information supplied by the authors. Such materials are peer reviewed and may be re‐organized for online delivery, but are not copy‐edited or typeset. Technical support issues arising from supporting information (other than missing files) should be addressed to the authors.

Supplementary
