# Supplementary material for: Matrix Effects in a Fluid Catalytic Cracking Catalyst Particle: Influence on Structure, Acidity, and Accessibility
Source: Chemistry. 2020 Aug 12;26(52):11995–2009. doi: 10.1002/chem.201905867 (PMC7539955; doi:10.1002/chem.201905867)
Supplement: Supplementary file 1 — Supplementary [file CHEM-26-11995-s001.pdf]

Chemistry–A European Journal

Supporting Information

Matrix Effects in a Fluid Catalytic Cracking Catalyst Particle: Influence on Structure, Acidity, and Accessibility

Marjolein E. Z. Velthoen,[a] Alessandra Lucini Paioni,[b] Iris E. Teune,[a] Marc Baldus,[b] and Bert M. Weckhuysen*[a]

Matrix Effects in a Fluid Catalytic Cracking Catalyst Particle: Influence on Structure, Acidity, and Accessibility

Marjolein E.Z. Velthoen,[a] Alessandra Lucini Paioni,[b] Iris E. Teune, [a] Marc Baldus[b] and Bert M. Weckhuysen*[a]

Supporting Information

a) Temperature-Programmed Desorption of NH_3

Figure S1 illustrates the TPD profiles for the six samples under study and indicates the band positions. The band around 400°C is assigned to NH_3 desorbing from Brønsted acid sites.[1] These Brønsted acid sites are inherent to the zeolite component and can, hence, be found in the Zeolite, ZeBi, and FCC samples. The assignment is rationalized by the absence of this band in the samples without a zeolite; i.e., the Binder, Clay, and BiC samples. The Brønsted acid sites of the zeolite appear to weaken in strength upon mixing with the binder and zeolite, as evidenced from the shift of the band maxima from 400 °C (zeolite sample) to 360 °C (ZeBi and FCC samples). Furthermore, the band at 200 °C is assigned to NH_3 desorbing from Lewis acid sites with a relatively weak nature.[1,2] Strong Lewis acid sites are not necessarily excluded in the samples, but due to the broad nature of the bands in **Figure S1**, it is difficult to unambiguously observe them in the TPD profiles, since they are hidden in the NH_3-TPD profile high-temperature tails.[3]

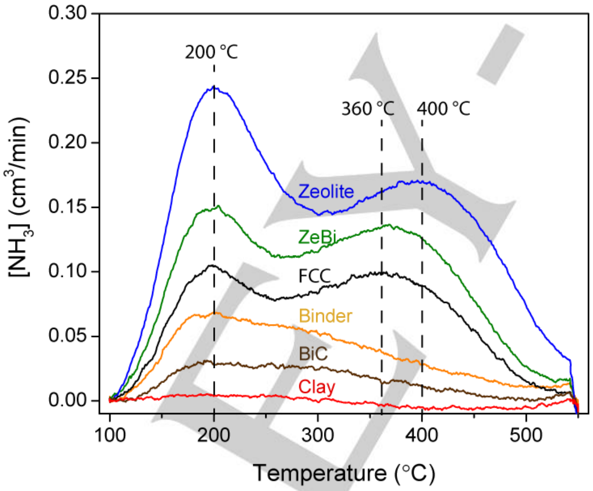

Figure S1. NH_3-TPD results for the six samples under study: single components Zeolite (black), Binder (magenta), and Clay (marine) and the spray-dried ZeBi (red), BiC (green), and FCC (blue) samples. The band at 200 °C is assigned to NH_3 coordinated to weak acid sites and the band between 360-400 °C is assigned to NH_3 adsorbed on Brønsted acid sites.

b) FT-IR Spectroscopy of CO Adsorption

Figure S2a and S2b illustrate the FT-IR spectra of the six samples before (black) and after CO adsorption (red) in the OH and CO vibrational region, respectively. In the CO vibrational region (2250-2050 cm^{-1}), the blue-shift of the original CO stretching vibration (i.e., 2143 cm^{-1} in its liquid state) upon interaction with an acid site, can be taken as a measure of the acidic strength and nature. By looking at the corresponding consumption of the bands in the OH vibrational region (3800-3500 cm^{-1}), a structure-acidity correlation can be established.[4–7]

In Figure S2b, the FCC catalyst displays two intense bands at 2164 and 2175 cm^{-1} that are attributed to CO adsorbed on weak and strong Brønsted acid sites, respectively. The appearance of these bands corresponds to the respective consumption of the bands at around 3600 and 3625 cm^{-1} in Figure S2a, which can be assigned to two types of Brønsted acid sites in the supercages of the zeolite.[8–10] The isolated silanol groups, characterized with a band at 3745 cm^{-1}, are also consumed upon CO adsorption. The interaction of CO with these silanol groups is usually signified by a band around 2158 cm^{-1}, as is the case for the binder and BiC sample.[11] The presence of this band cannot be confirmed in the FCC spectrum due to the overlap of adjacent intense bands at 2164 and 2143 cm^{-1}.

WILEY-VCH

In addition to Brønsted acid sites, there are also Lewis acid sites present in the FCC catalyst, as evidenced by the presence of bands at 2230 and 2195 cm^{-1} corresponding to CO adsorbing on strong and weak Lewis acid sites, respectively.[11,12] Together with the strong Brønsted acid sites, these Lewis acid sites are the first ones to interact with CO upon the first pulse. This is clearly visualized in Figure S2c and S2d, showing the OH and CO vibrational region, respectively, before (black) and after the first CO pulse (blue). Three bands are visible that can be assigned to the CO stretching vibration when adsorbed on different acid sites. The band at 2180 cm^{-1} corresponds to the strong Brønsted acid sites. At higher CO loadings, this band shifts to 2175 cm^{-1}.[7] The appearance of the two bands at 2230 and 2195 cm^{-1}, corresponding to strong and weak Lewis acid sites, does not seem to be accompanied by the direct consumption of a hydroxyl group band. This is expected, since hydroxyl groups are not Lewis acid sites, but Al centers are. Interestingly, however, the OH vibrational bands at 3695 and 3745 cm^{-1}, assigned to aluminol and silanol groups respectively, show a significant blue-shift upon the interaction of CO with Lewis acid sites. When CO donates electron density into the empty orbital of the Lewis acidic aluminum center, this transfer is possibly stabilized by transferring some of the electron density into the neighboring O-H bond. This strengthens the O-H bond, causing a blue-shift in the FT-IR spectrum. The indirect indications of Lewis acidity via the observed perturbation of hydroxyl groups has been demonstrated before by Busca and co-workers.[8] This observation can provide important information on the structural origin of Lewis acid sites in the FCC catalyst, as it indicates that Lewis acidic Al sites in the FCC catalyst are in the near proximity of hydroxyl groups. In particular, Figure S2 demonstrates that the Lewis acid sites near silanol groups (3742 cm^{-1}) are inherent to the zeolite and binder domains, whereas strong Lewis acid sites near aluminol groups can originate from the clay (3695 and 3675 cm^{-1}) or the zeolite (3670 cm^{-1}).

The clay component demonstrates four distinct bands in the O-H vibrational region at 3700, 3675, 3660, and 3620 cm^{-1}, which

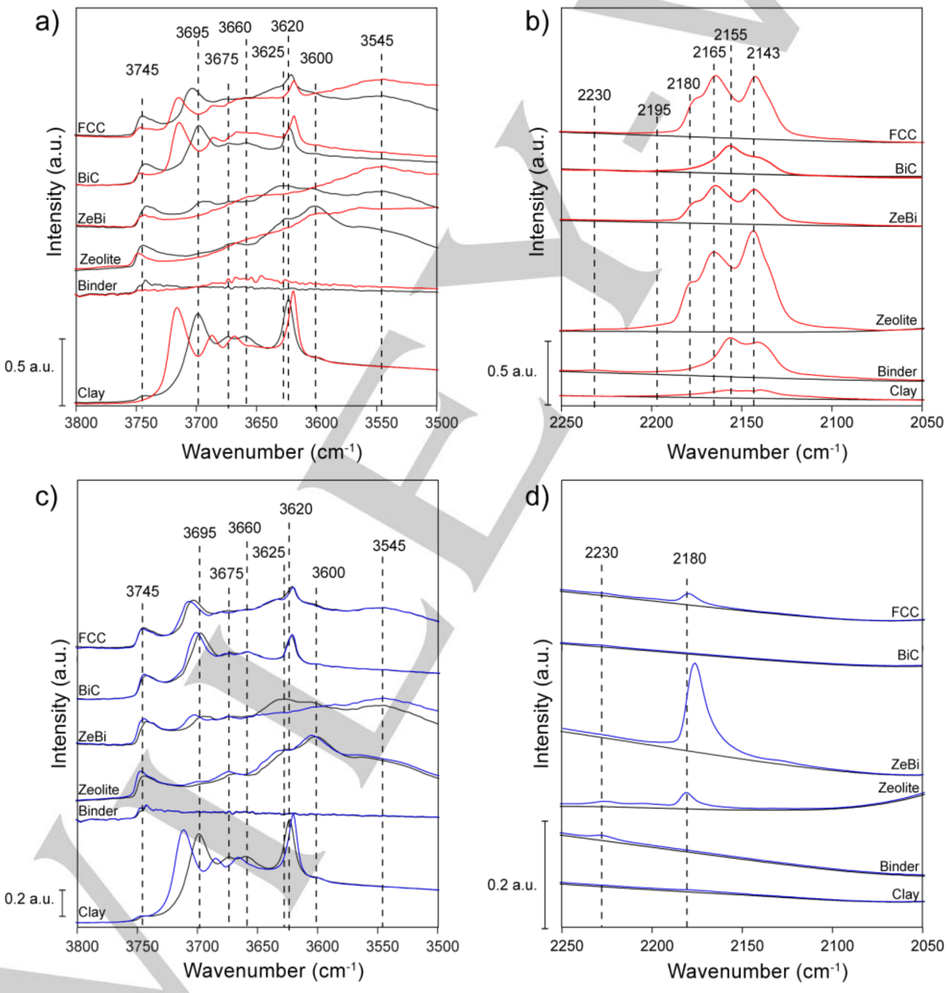

Figure S2. FT-IR spectra of the spray dried FCC, BiC, and ZeBi samples and corresponding individual components Zeolite, Binder, and Clay after heat-treatment at 550 °C for 1 h under dynamic vacuum (black) and after subsequent CO adsorption at -188 °C (red). Figures a/c) and b/d) show the FT-IR spectra in the OH and CO vibrational region, respectively. Figures a/b) show the spectra before and after full CO loading. Figures c/d) depict the spectra before and after minimal CO loading. The latter spectra, therefore, indicate the strongest acid sites present in the FCC catalyst.

correspond to two types of hydroxyl groups. The first three absorption bands are the result of the coupling of three inner surface hydroxyl groups to give one in-phase vibration at 3700 cm^{-1} and two anti-phase vibrations at 3675 and 3660 cm^{-1}.[13] The absorption at 3620 cm-

1 is affiliated with a hydroxyl group bound to the tetrahedral sheet within the kaolin clay. A small shoulder at 3600 cm^{-1} can be observed, which was ascribed to Brønsted acidity in the FCC catalyst. There is, indeed, a hint of Brønsted acidity evidenced from an absorption band at 2180 cm^{-1}. This is also the first band to arise upon minimal loading of CO. The clay, however, does not express high levels of acidity, as indicated by the overall low absorbance in Figure S2b, but this is often ascribed to the fact that kaolin is very dense and not accessible.[2] Furthermore, Figure S2b demonstrates some physisorbed CO at 2143 cm^{-1} and some CO adsorbed on hydroxyl groups around 2155 cm^{-1}. Interestingly, the CO vibrational region does not indicate the clear presence of Lewis acid sites, but there are other indications of Lewis acidity. Directly upon the adsorption of CO, the first three hydroxyl groups express strong blue-shifts, as observed in Figure S2c. These shifts indicate the donation of electron density from the CO to the hydroxyl groups. The capability of accepting this electron density is by definition Lewis acidity. Based on these results, the kaolin clay appears to possess Lewis acid sites.

The binder barely contains surface hydroxyl groups, except for some silanol groups at 3745 cm^{-1}. Upon CO adsorption, the silanol groups are consumed and the perturbed O-H vibration is observed around 3660 cm^{-1}. With minimal CO loading, however, a clear absorption band at 2230 cm^{-1} appears, indicating the presence of strong Lewis acid sites. At maximum CO loading, we also evidence the presence of weaker Lewis acid sites (2195 cm^{-1}), CO adsorbed on silanol groups (2155 cm^{-1}), physisorbed CO (2143 cm^{-1}) and a small amount of weak Brønsted acidity at 2165 cm^{-1}, indicating that the boehmite and silica have interacted during calcination and formed a mixed silica/alumina phase with Brønsted acid sites.[14]

CO FT-IR spectroscopy experiments for the zeolite express similar spectral features as for the FCC catalyst. The adsorption of the first CO molecules is evidenced from absorption bands at 2230 cm^{-1} and 2180 cm^{-1}, corresponding to strong Lewis acid sites and strong Brønsted acid sites, respectively. With increasing CO loading, two types of Brønsted acid sites can be distinguished at 2180 and 2165 cm^{-1}, affiliated with the consumption of the Brønsted acidic OH groups at 3625 and 3600 cm^{-1}, respectively. Similar to what was observed for the clay component, the adsorption of CO on the Lewis acid sites seems to be affiliated with the blue-shift of some O-H vibrations. The affected hydroxyl groups are both aluminol groups (3670 cm^{-1}) and Brønsted acidic hydroxyl groups (3625, 3600, and 3560 cm^{-1}). This indicates that zeolitic framework Al species that are associated with Brønsted acidity can possibly also function as a Lewis acid site.[8] Interestingly, one can imagine that when this Al center actually acts as a Lewis acid site, part of the accepted electron density is transferred to the bridging O-H group, weakening the connected Brønsted acid site.

The interaction between the zeolite and binder components in the spray-dried ZeBi sample is visualized in Figure S2 as well, showing similar sites as found in the zeolite and binder sample. Importantly, however, the ZeBi sample does not seem to contain strong Lewis acid sites (2230 cm^{-1}) anymore, indicating an interaction between the two components and confirming the results from NH_3-TPD. Additionally, the band for strong Brønsted acid sites around 2175 cm^{-1} in the zeolite component seems red-shifted to lower wavenumbers. This indicates that the Brønsted acid sites in the ZeBi sample have weakened due to the interaction with the binder component.[6] Finally, the ZeBi sample contains a clear band at 3695 cm^{-1} that is not clearly present in either the zeolite or the binder. This band is assigned to isolated Al-OH groups that are probably formed upon the interaction of the CUS in aqueous solution during the synthesis of this sample.[5] Upon the first interaction with CO, this band blue-shifts, indicating that the CO molecules act as Lewis bases, donating electron density to the Lewis acidic Al center (Figure S2c). At maximum CO loading, however, the band is fully consumed, indicating that CO can also adsorb on the hydroxyl groups themselves. Except for a clear dilution of the acidic properties, the spectral features for the BiC sample simply seem to be the average of the individual two components. Interestingly, the strong Lewis acid sites (2230 cm^{-1}) in the binder are preserved upon interaction with the clay component, whereas these had disappeared upon the interaction between binder and zeolite. This either implies that the clay prevents the full removal of strong Lewis acid sites in the FCC catalyst as a result of zeolite-binder interactions or it means that the strong Lewis acid sites in the FCC catalyst all originate from the clay component.

c) Argon Adsorption

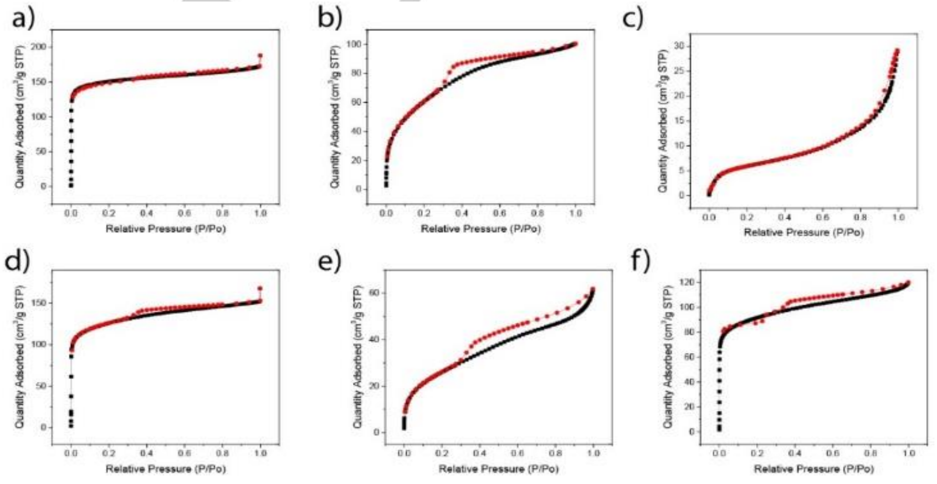

Figure S3. Ar adsorption (black) and desorption (red) isotherms for zeolite (a), binder (b), clay (c), ZeBi (d), BiC (e), and FCC (f).

d) X-ray Diffraction

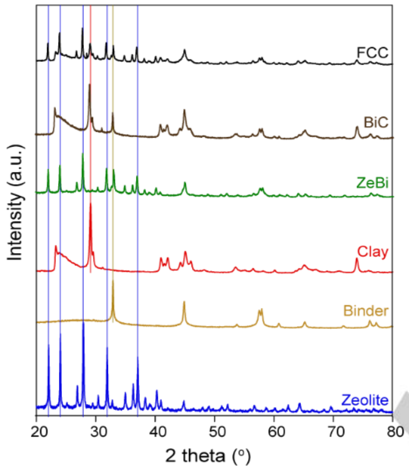

Figure S4. X-ray Diffraction (XRD) patterns of the individual components: zeolite, binder, and clay, and of the spray-dried samples ZeBi, BiC, and FCC. The colored vertical bars indicate from which components originate the most intense peaks in the spray-dried samples.

e) FT-IR spectroscopy

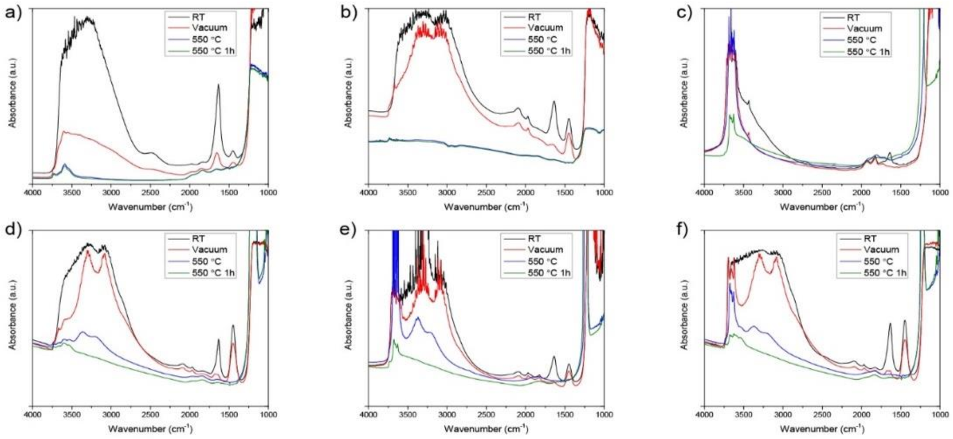

Figure S5. FT-IR spectra of the six samples under study at room temperature (black), after vacuum desorption for 45 min (red), after heating to 550 °C with 10 °C/min (blue), and after holding the sample another hour at 550 °C (green): zeolite (a), binder (b), clay (c), ZeBi (d), BiC (e), and FCC (f).

References

[1] R. Oord, I. C. Ten Have, J. M. Arends, F. C. Hendriks, J. Schmidt, I. Lezcano-Gonzalez, B. M. Weckhuysen, *Catal. Sci. Technol.* **2017**, *7*, 3851–3862.

[2] G. T. Whiting, A. D. Chowdhury, R. Oord, P. Paalanen, B. M. Weckhuysen, *Faraday Discuss.* **2016**, *188*, 369–386.

[3] F. Lónyi, J. Valyon, *Microporous Mesoporous Mater.* **2001**, *47*, 293–301.

[4] T. H. Ballinger, J. T. Yates, *Langmuir* **1991**, *7*, 3041–3045.

[5] H. Knözinger, in *Elem. React. Steps Heterog. Catal.* (Eds.: R.W. Joyner, R.A. van Santen), Springer, Dordrecht, **1993**, pp. 267–285.

[6] H. Knözinger, in *Handb. Heterog. Catal.* (Eds.: G. Ertl, F. Schuth, H. Knözinger, J. Weitkamp), Wiley-VCH, Weinheim, **2008**, pp. 1135–1162.

[7] S. Bordiga, C. Lamberti, F. Bonino, A. Travert, F. Thibault-Starzyk, T.-S. Frédéric, F. Thibault-Starzyk, *Chem. Soc. Rev.* **2015**, *44*, 7262–7341.

[8] T. K. Phung, G. Busca, *Appl. Catal. A Gen.* **2015**, *504*, 151–157.

[9] E. G. Derouane, J. C. Védrine, R. Ramos Pinto, P. M. Borges, L. Costa, M. A. N. D. A. Lemos, F. Lemos, F. Ramôa Ribeiro, *Catal. Rev. Sci. Eng.* **2013**, *55*, 454–515.

[10] S. Khabtou, T. Chevreau, J. C. Lavalley, *Microporous Mater.* **1994**, *3*, 133–148.

[11] A. Vimont, F. Thibault-Starzyk, M. Daturi, *Chem. Soc. Rev.* **2010**, *39*, 4928–4950.

[12] M. I. Zaki, H. Knözinger, *Mater. Chem. Phys.* **1987**, *17*, 201–215.

[13] V. C. Farmer, *Clay Miner.* **1998**, *33*, 601–604.

[14] M. Caillot, A. Chaumonnot, M. Digne, J. A. van Bokhoven, *J. Catal.* **2014**, *316*, 47–56.
